# High-throughput characterization of the effect of sodium chloride and potassium chloride on 31 lactic acid bacteria and their co-cultures

**DOI:** 10.3389/fmicb.2024.1328416

**Published:** 2024-02-16

**Authors:** Amadou Ndiaye, Ismail Fliss, Marie Filteau

**Affiliations:** ^1^Département des Sciences des Aliments, Université Laval, Québec, QC, Canada; ^2^Institut sur la Nutrition et les Aliments Fonctionnels (INAF), Québec, QC, Canada; ^3^Institut de Biologie Intégrative et des Systèmes (IBIS), Université Laval, Québec, QC, Canada

**Keywords:** lactic acid bacteria, microbial interactions, salt, high-throughput culturing, image analysis, acidification, bacterial co-culture

## Abstract

Salt (NaCl) is associated with a risk of hypertension and the development of coronary heart disease, so its consumption should be limited. However, salt plays a key role in the quality and safety of food by controlling undesirable microorganisms. Since studies have focused primarily on the effect of salts on the overall counts of the lactic acid bacteria (LAB) group, we have not yet understood how salt stress individually affects the strains and the interactions between them. In this study, we characterized the effect of sodium chloride (NaCl) and potassium chloride (KCl) on the growth and acidification of 31 LAB strains. In addition, we evaluated the effect of salts on a total of 93 random pairwise strain combinations. Strains and co-cultures were tested at 3% NaCl, 5% NaCl, and 3% KCl on solid medium using an automated approach and image analysis. The results showed that the growth of LAB was significantly reduced by up to 68% at 5% NaCl (*p* < 0.0001). For the co-cultures, a reduction of up to 57% was observed at 5% NaCl (*p* < 0.0001). However, acidification was less affected by salt stress, whether for monocultures or co-cultures. Furthermore, KCl had a lesser impact on both growth and acidification compared to NaCl. Indeed, some strains showed a significant increase in growth at 3% KCl, such as *Lactococcus lactis* subsp. *lactis* 74310 (23%, *p* = 0.01). More importantly, co-cultures appeared to be more resilient and had more varied responses to salt stress than the monocultures, as several cases of suppression of the significant effect of salts on acidification and growth were detected. Our results highlight that while salts can modulate microbial interactions, these latter can also attenuate the effect of salts on LAB.

## Introduction

The use of salt, which dates back to the Neolithic era, is used in many foods for its technological functionalities related to texture or taste, but most importantly for preservation, through the inhibition of pathogenic or spoilage microorganisms (Albarracín et al., 2011; Fraqueza et al., 2021). In food products, the use of sodium chloride (NaCl) helps reduce the proliferation of undesirable microorganisms, which can sometimes include lactic acid bacteria (LAB) (Muruzović et al., 2018; Bansal and Mishra, 2020). However, high salt intake is associated with increased blood pressure, which can lead to coronary heart disease, stroke, and other chronic diseases (Farquhar et al., 2015; Takase et al., 2023). This negative impact of salt on human health has led public health organizations to recommend a reduction in its consumption, of which 77% of intake comes from processed foods, including cheese, in Canada (Health Canada, 2018). Furthermore, consumers are increasingly concerned about the quality of food products, including the amount of salt they contain (Petit et al., 2019; Sun et al., 2021).

To address this issue, several strategies have been explored, including reducing the salt content in prepared foods. However, reducing the amount of salt in foods poses challenges to product quality and safety (Ganesan et al., 2014; Bansal and Mishra, 2020). For instance, in cheese made from pasteurized milk and intentionally inoculated with contaminants, studies have shown that salt reduction can increase the survival of undesirable bacteria such as *Pseudomonas fragi* (Dugat-Bony et al., 2016) or *Salmonella* (Shrestha et al., 2011) and can promote the proliferation of LAB, resulting in increased proteolysis that can lead to a bitter taste (Rulikowska et al., 2013). The same issue of increased spoilage bacteria has been observed in meat products (Fougy et al., 2016).

An alternative to preserve consumer health while ensuring food quality is the partial or total substitution of NaCl with other salts such as potassium chloride (KCl), magnesium chloride (MgCl_2_), and calcium chloride (CaCl_2_). Among these candidates, KCl appears to stand out, as it has been shown to play a role in reducing blood pressure (Ekmekcioglu et al., 2016; Bernabe-Ortiz et al., 2020), but this might be an issue for people with chronic kidney disease (Morrison et al., 2021). Furthermore, at high concentrations, KCl imparts a bitter metalic taste to food products, leading to lower consumer acceptability (Khetra et al., 2016). Although in some studies, the substitution of NaCl with KCl had no significant impact on the growth of LAB (Ayyash and Shah, 2010; Kamleh et al., 2012; Juan et al., 2022), others showed that this change promotes their growth (Karimi et al., 2012). However, differences at the strain level could lead to this discrepancy which raises questions about how the effect of salts varies among LAB strains in terms of growth and metabolic response, for instance, their acidification ability (Papadopoulou et al., 2023).

LAB play several important roles in our diet. They are fundamental in fermentation and flavor development in various food products (Gänzle, 2015; Hu et al., 2022). Additionally, LAB act as bioprotective cultures because of their ability to produce molecules that can inhibit the growth of pathogens or spoilage microorganisms. For instance, the strain *Carnobacterium divergens* M35 produces a bacteriocin with antilisterial activity for ready-to-eat seafood products (Tahiri et al., 2004, 2009). Moreover, LAB are a preferred group of probiotics, as they can positively improve and balance the intestinal microbiota (Anjana and Tiwari, 2022). However, lactic acid bacteria can also be responsible for food spoilage (Pothakos et al., 2014; Hultman et al., 2015). Therefore, a better understanding of LAB at the individual level and in the context of co-culture is relevant to improve their use or control in food products (Andreevskaya et al., 2018).

LAB are often used in mixed cultures to obtain desired characteristics, due to their ability to adapt to environmental variations and their versatility to perform complex metabolic activities compared to pure cultures (Smid and Lacroix, 2013). This community life leads to microbial interactions between them, but also with other food exogenous microorganisms. The importance of microbial interactions in complex ecosystems is well established, as their outcomes in terms of functions, growth, and fitness can shape their environment (Gupta et al., 2021). On the basis of this fact, considering acidification and not just growth in an interaction context is highly relevant for LAB. Therefore, the hypothesis that salt stress could influence microbial interactions and their functional outcomes should not be overlooked.

The aim of this study was to measure the effect of NaCl and KCl on LAB at the strain level as well as in pairwise co-cultures. We implemented a high-throughput approach to monitor bacterial cultures on solid media using a colony handling robot and image analysis. The growth and acidification of 31 LAB strains and 93 binary combinations were quantified. Our results demonstrate that salt generally reduces the growth of LAB, but its impact on acidification is more variable between strains. Moreover, our findings indicate that co-cultures can mitigate the impact of salts on LAB and that salts have the potential to influence microbial interactions. Taken together, our results underline the relevance of investigating individual strains and microbial interactions in the context of LAB applications in the food industry.

## Materials and methods

### Bacterial strains and cultivation

The 31 lactic acid bacteria strains used in this study come from the Réseau des bactéries lactiques (RBL) collection held at Université Laval (Table 1). The selected strains belong to species of interest for food applications such as cheese production or food bioprotection applications. For liquid growth, plate count broth supplemented with skim milk (PCBS) containing 0.5% tryptone, 0.25% yeast extract, 0.1% glucose, and 0.1% skim milk was used. For plate growth, PCBS was supplemented with 2% agar (PCAS) and 0.1% litmus (PCASL) as a pH indicator. Different salt concentrations (3% NaCl, 5% NaCl, and 3% KCl) were added where indicated. To prepare precultures from glycerol stock, all strains were first grown in PCBS and incubated at 30°C for 48 h. Cultures were then centrifuged at 5000 rpm for 5 min at 4°C and 50% of the supernatant volume was removed. Bacterial cultures were vortexed and 150 μL were transferred to a 96-well plate. With a colony handling robot featuring a 96-pin tool (BM3-BC, S&P Robotics, Inc., North York, ON, Canada), bacterial cells were transferred in 96-spot format on OmniTray™plates on PCAS medium (35 mL) and incubated at 20°C for 4 days.

**Table 1:** List of strains used in this study.

Microorganisms	Strain number	Abbreviation	Isolation source
*Carnobacterium divergens*	M35	C.divergens	Smoked mussels
*Lacticaseibacillus paracasei*	ATCC 334	Lc.paracasei	Emmental cheese
*Lacticaseibacillus rhamnosus*	ATCC 8530	Lc.rhamnosus	Human gastrointestinal tract
	ATCC 10863	Lc.rhamnosus	N/A
	ATCC 13075	Lc.rhamnosus	Ripening Swiss cheese
	ATCC 27773	Lc.rhamnosus	Derived from existing strain
*Lactiplantibacillus pentosus*	ATCC 8041	Lp.pentosus	Corn silage
*Lactobacillus delbrueckii* subsp. *bulgaricus*	ATCC 11842	Lb.bulgaricus	Bulgarian yogurt
	UL12	Lb.bulgaricus	Cheddar cheese
*Lactobacillus helveticus*	ATCC 15009	Lb.helveticus	Emmental cheese
*Lactococcus cremoris*	7308	L.cremoris	Dairy starter
	7311	L.cremoris	Dairy starter
	7313	L.cremoris	Dairy starter
	7322	L.cremoris	Dairy starter
	7328	L.cremoris	Dairy starter
	ATCC 19257	L.cremoris	Cheese
	KB	L.cremoris	Commercial starter
*Lactococcus lactis* subsp. *lactis*	1919	L.lactis	Dairy starter
	11121	L.lactis	Dairy starter
	29828	L.lactis	Dairy starter
	74308	L.lactis	Dairy starter
	74309	L.lactis	Dairy starter
	74310	L.lactis	Dairy starter
	IL 1403	L.lactis	Derivative of the strain IL594, isolated from a cheese starter culture
	LM 0230	L.lactis	N/A
	MJC 5	L.lactis	Raw milk cheese
	MJC 8	L.lactis	Raw milk cheese
*Lactococcus lactis* subsp. *lactis* bv. *diacetylactis*	UL 719	L.diacetylactis	Raw milk cheese
*Lactococcus raffinolactis*	ATCC 43920	L.raffinolactis	Raw milk
*Pediococcus pentosaceus*	ATCC 33316	P.pentosaceus	Dried beer yeast
*Streptococcus thermophilus*	FYE 41	S.thermophilus	Commercial starter

### Salts and microbial interaction assays

**Figure 1:**
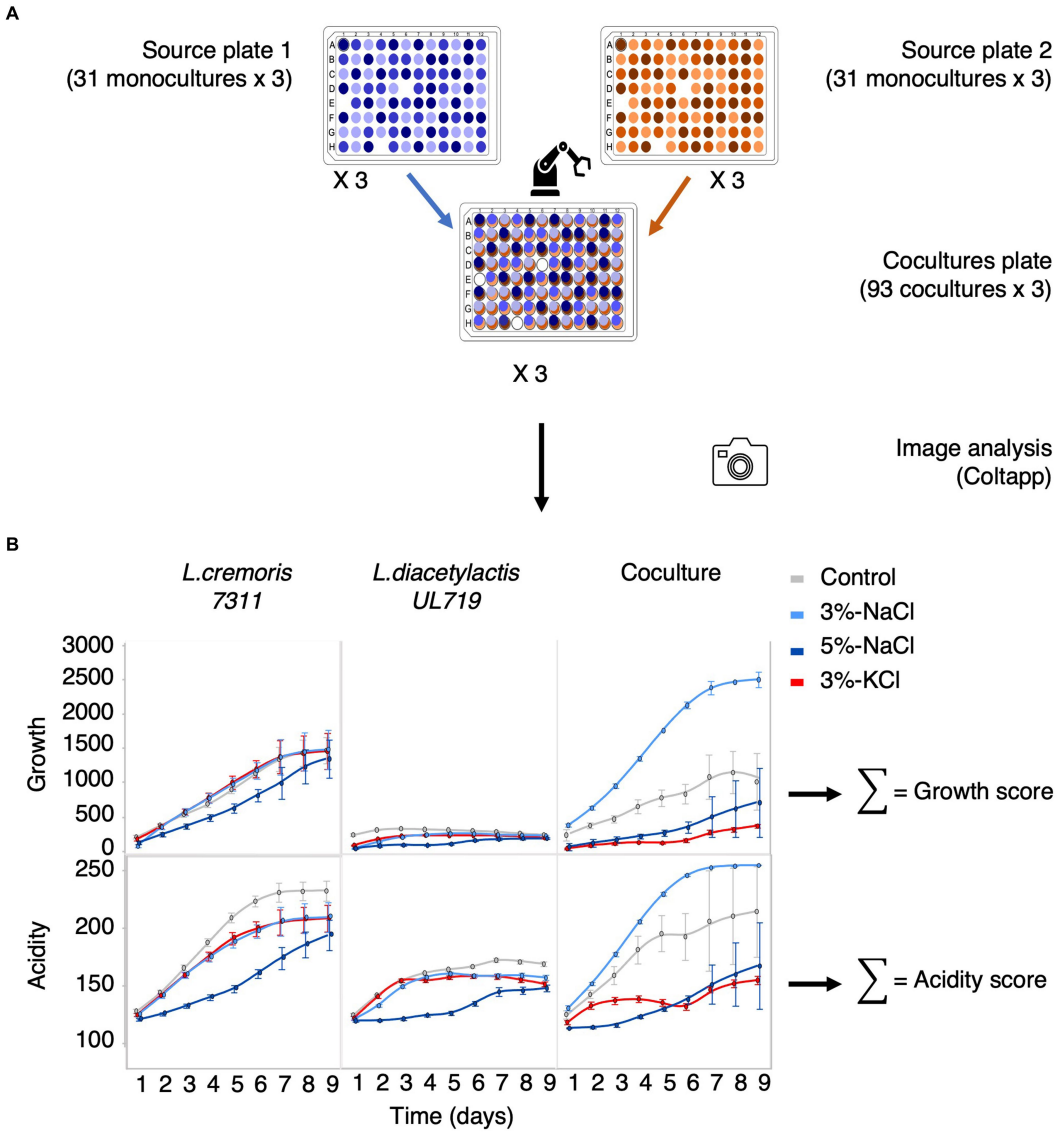
Measuring growth and acidification of pure cultures and their binary co-cultures using a high-throughput approach. **(A)** Schematic setup of the experiment: two 96-spot plates, each containing pure cultures, are combined by the robot to form a 96-spot plate with co-cultures at each spot (93 co-cultures and three negative controls). **(B)** Growth and acidification are quantified using image analysis (Coltapp). For instance, tracking the growth and acidification of *Lactococcus cremoris 7311*, *Lactococcus lactis* subsp. *diacetylactis UL 719*, and their co-culture is performed over a period of 9 days. The light gray curve represents the control, while the light blue, dark blue, and red curves represent 3% NaCl, 5% NaCl, and 3% KCl, respectively. The growth score reflects the cumulative biomass based on the size and opacity of the colony. The acidity score corresponds to the cumulative red intensity on the RGB scale.

Two 96-position plate designs (plate 1 and 2) were constructed with the 31 strains, each randomized at six different positions (three positions per plate), leaving three empty positions as negative controls (Figure 1A, Supplementary Table 1). Then each plate was replicated on PCASL medium and on PCASL supplemented with 3% NaCl, 5% NaCl, or 3% KCl. Each plate and condition were produced in triplicate for a total of 24 monoculture plates. To assess the impact of microbial interactions, co-cultures were created by pinning plates 1 and 2 on top of each other, resulting in 93 pairs on the interaction plate. This design was replicated three times on each culture medium (PCASL as the controls and PCASL with 3% NaCl, 5% NaCl, or 3% KCl as the treatments). All plates were incubated at 20°C for 10 days. The automated platform was equipped with a camera (Canon EOS Rebel T7i) to capture images of OmniTray™ plates. The first images were taken after 24 h of incubation at 20°C and subsequently at regular intervals during the day for 9 days for a total of 18 time points.

### Quantification of growth and acidification through image analysis

Images were processed using ColTapp, a software application designed to quantify the growth dynamics of microbial colonies and other related metrics (Bär et al., 2020). The process first involved applying automatic colony detection to a late reference photograph, then detecting colonies in other photographs of the time-lapse and tracking their radius over time. Default settings were used, except for the maximum radius, which was changed to 90 pixels, and the maximum overlap (2 circles), which was set to 0.1. After manual adjustments to improve the accuracy of colony detection, data on growth and acidity changes were exported at each time point (Figure 1B, Supplementary Table 2). Microbial growth was quantified using the product of colony size (radiusum expressed in micrometers) and colony opacity (MeanGray expressed as grayscale color value between 0 and 1). We calculated a growth score corresponding to the sum of these values, that is, the growth score is an estimate of the integrated microbial biomass. Acidification was monitored by adding litmus to the medium that changes from blue to red below pH 4.5. An acidity score was calculated by using the sum of MeanRed color values (intensity of red color expressed in RGB value between 0 and 255) for each colony over the time-lapse. The more intense and rapid the acidification, the higher the acidification score.

### Statistical analysis

Statistical analysis was performed using JMP Pro version 16.2. Each strain was tested with a total of 18 replicates for the monoculture condition and three replicates for the interaction condition. To compare the average effects on all strains, a t-test was used to compare the control and salts treatments. The Pearson correlation was used to report on the relationship between growth score and acidity score, excluding outliers identified with the Robust Fit (Huber) method. With unequal variances (Bartlett’s test<0.0001) when comparing the monoculture group with the co-culture group, we used a Welch’s test to assess whether the response of the co-cultures was significantly different from that of the monocultures. To assess the difference between the control and salt treatments for each strain or co-culture, the standard least squares method was employed to fit the linear model in ANOVA, followed by the application of the Dunnett *post hoc* test. The resulting *p*-values values were subjected to a false discovery rate (FDR) adjustment. To analyze whether microbial interactions varied between culture media we employed a mixed model and estimated the parameters of the model using the restricted maximum likelihood method (REML). The position of the strains on the plates served as a random effect and the fixed effects were the composition of the medium (Salt effect) and the mono/co-culture condition (Co-culture effect). *p*-values were adjusted with the FDR method.

## Results

### Salt effects on LAB strains

To characterize the effects of salt on 31 LAB strains, we performed a high-throughput assay on solid media using a colony handling platform and quantified growth and acidification through image analysis. We obtained a growth score reflecting the cumulative biomass over time and an acidification score based on the cumulative color change of a pH indicator in the medium over time. These two scores were calculated for each strain under a control condition and at different salt concentrations (3% NaCl, 5% NaCl, and 3% KCl).

**Figure 2:**
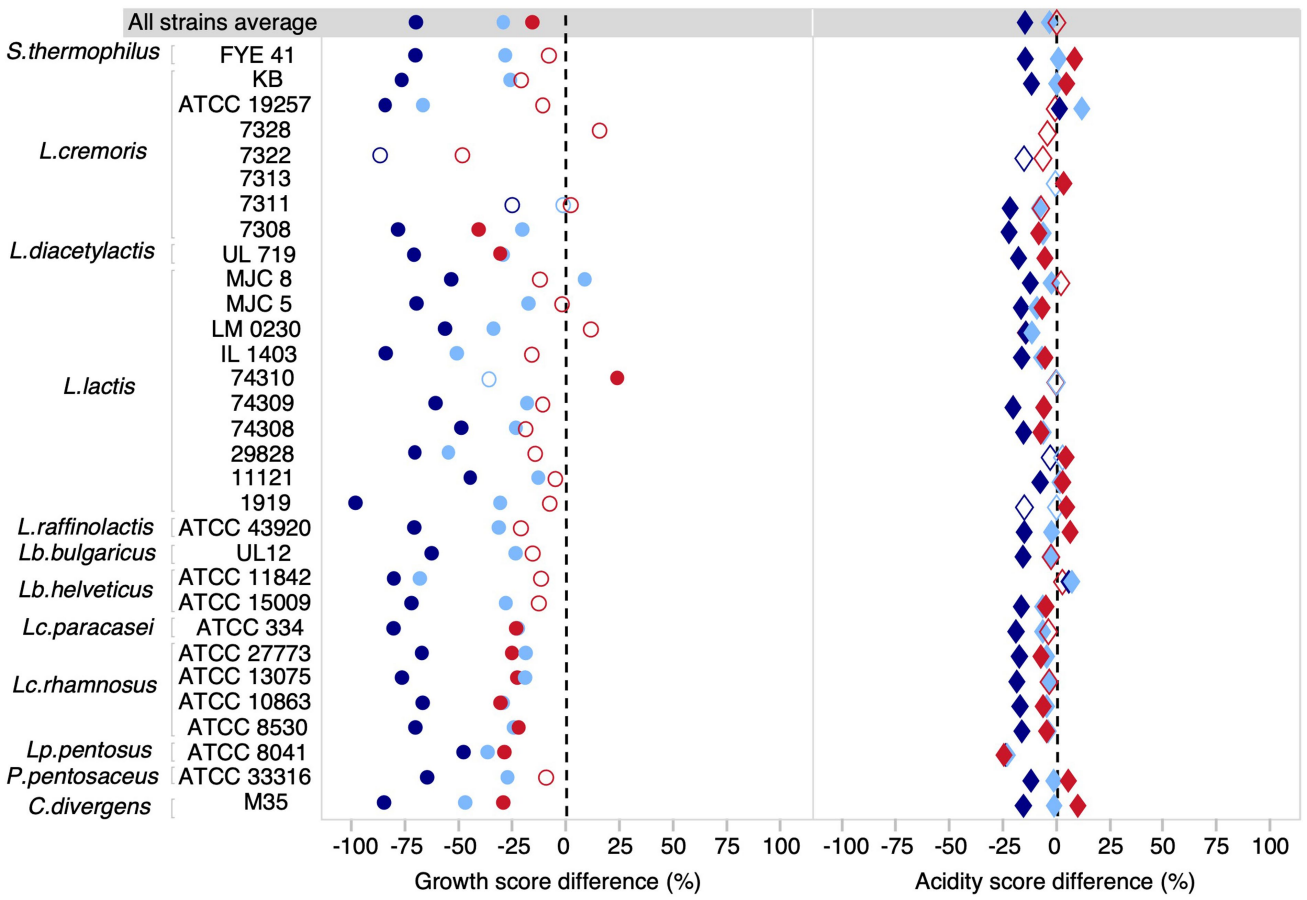
Difference of growth and acidification scores between each salt condition (3% NaCl, 5% NaCl, and 3% KCl) relative to the control (PCASL). Difference of growth and acidification score for each salt condition. The growth score reflects the cumulative biomass based on the size and opacity of the colony. The acidity score corresponds to the cumulative red intensity on the RGB scale. Light blue, dark blue, and red shapes represent differences at 3% NaCl, 5% NaCl, and 3% KCl concentrations, respectively. Filled shapes indicate statistically significant differences (FDR adjusted *P-*value < 0.05), while hollow shapes were non-significant.

We first examined the average effects for our community of 31 strains. In general, the different salt conditions tested had less impact on acidification than on growth (Figure 2, Supplementary Table 3). The growth score was reduced on average by 69% at 5% NaCl (*t*-test, *p* < 0.0001), 29% for 3% NaCl (*t*-test, *p* < 0.0001) and 15% for a KCl concentration of 3% (*t*-test, *p* < 0.0001). For acidification, the average reduction was 14% at 5% NaCl concentration (*t*-test, *p* < 0.0001) and only 3% at 3% NaCl (*t*-test, *p* < 0.02). However, at 3% KCl, there was no statistically significant effect at the community level (*t*-test, *p* = 0.18). This highlights the importance of considering strain-level effects since the directionality may vary between strains and be obscured in community-level analysis. Indeed, when considering strains individually, the effects of NaCl were significant and negative for most strains, both for growth and acidification, but the effects of KCl were more variable (Figure 2). The negative effect of KCl on growth was significant for all *Lacticaseibacillus* strains, but not significant in *Lactococcus* strains, with the exception of *L. diacetylactis* UL719 and *L. cremoris* 7308. *L. lactis* 74310 even showed an increase in growth in the presence of 3% KCl. Furthermore, the effect of NaCl was not always linear with respect to concentration. For example, *Lactococcus lactis* subsp. *lactis* MJC 8 had a growth increase of 9% at 3% NaCl (Dunnett’s test, FDR *p* = 0,002) but a reduction of 53% at 5% NaCl (Dunnett’s test, FDR *p* = 0.002). Several strains had a higher acidification score under salt stress. For example, both NaCl concentrations tested had a positive effect on the acidification of *Lactobacillus delbrueckii subsp. bulgaricus* ATCC 11842. However, for some strains, such as *Streptococcus thermophilus* FYE 41, the positive effect on acidification appeared to be related to the concentration and type of salt, i.e., the effect was positive for 3% NaCl and 3% KCl, but negative for 5% NaCl. In general, higher acidification scores were most often observed for the KCl condition, while the 5% NaCl condition had the strongest negative impact for both measured parameters. It should be noted that *L. cremoris* 7322 and 7328 did not show quantifiable growth at 3% NaCl and that *L. cremoris* 7313, *L. cremoris* 7328, and *L. lactis* 74310 did not show growth at 5% NaCl.

**Figure 3:**
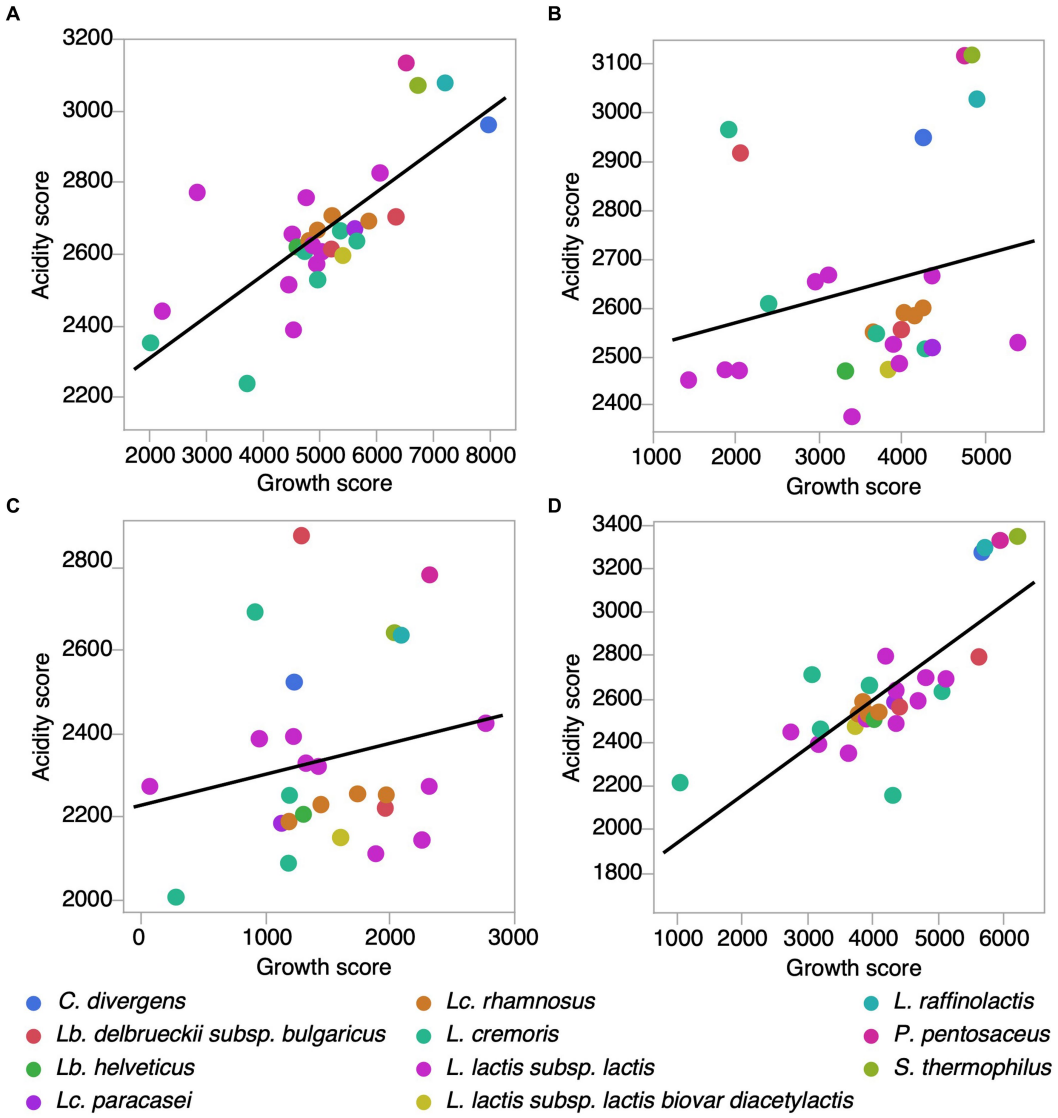
Correlation between acidification and growth scores for LAB strains in different salt conditions. **(A)** Control (PCASL media) (Pearson correlation *r* = 0.7394, *p* < 0.0001, *N* = 29). **(B)** 3% NaCl (Pearson correlation *r* = 0.2266, *p* = 0.25, *N* = 27). **(C)** 5% NaCl (*r* = 0.2061, *p* = 0.31, *N* = 26). **(D)** 3% KCl (Pearson correlation *r* = 0.7694, *p* < 0.0001, *N* = 29). Note that *Lactiplantibacillus pentosus* ATCC 8041 and *Lactoccocus cremoris* 7,311 were identified as growth score outliers and excluded from these analysis.

In the absence of added salt, acidification scores for individual strains were positively correlated with growth scores (Pearson correlation = 0.7394, *p* < 0.0001, *N* = 29) (Figure 3A, Supplementary Table 4). Similarly, in the presence of 3% KCl, this correlation was also significant (Pearson’s correlation = 0.7694, *p* < 0.0001, *N* = 29) (Figure 3D). However, at 3% NaCl and 5% NaCl, the correlation between acidification and growth was not significant (Pearson’s correlation 3% NaCl = 0.2266, *p* = 0.2557, *N* = 27; Pearson’s correlation 5% NaCl = 0.2061, *p* = 0.3125, *N* = 26) (Figures 3B,C). As *Lp. pentosus* ATCC 8041 and *L. cremoris* 7311 were identified as growth score outliers, they were excluded from this analysis. These results show that among strains, the relationship between acidification and growth, i.e., the metabolic strategies used for growth, can be modulated by the presence of salt.

### Salt effects on LAB co-cultures

**Figure 4:**
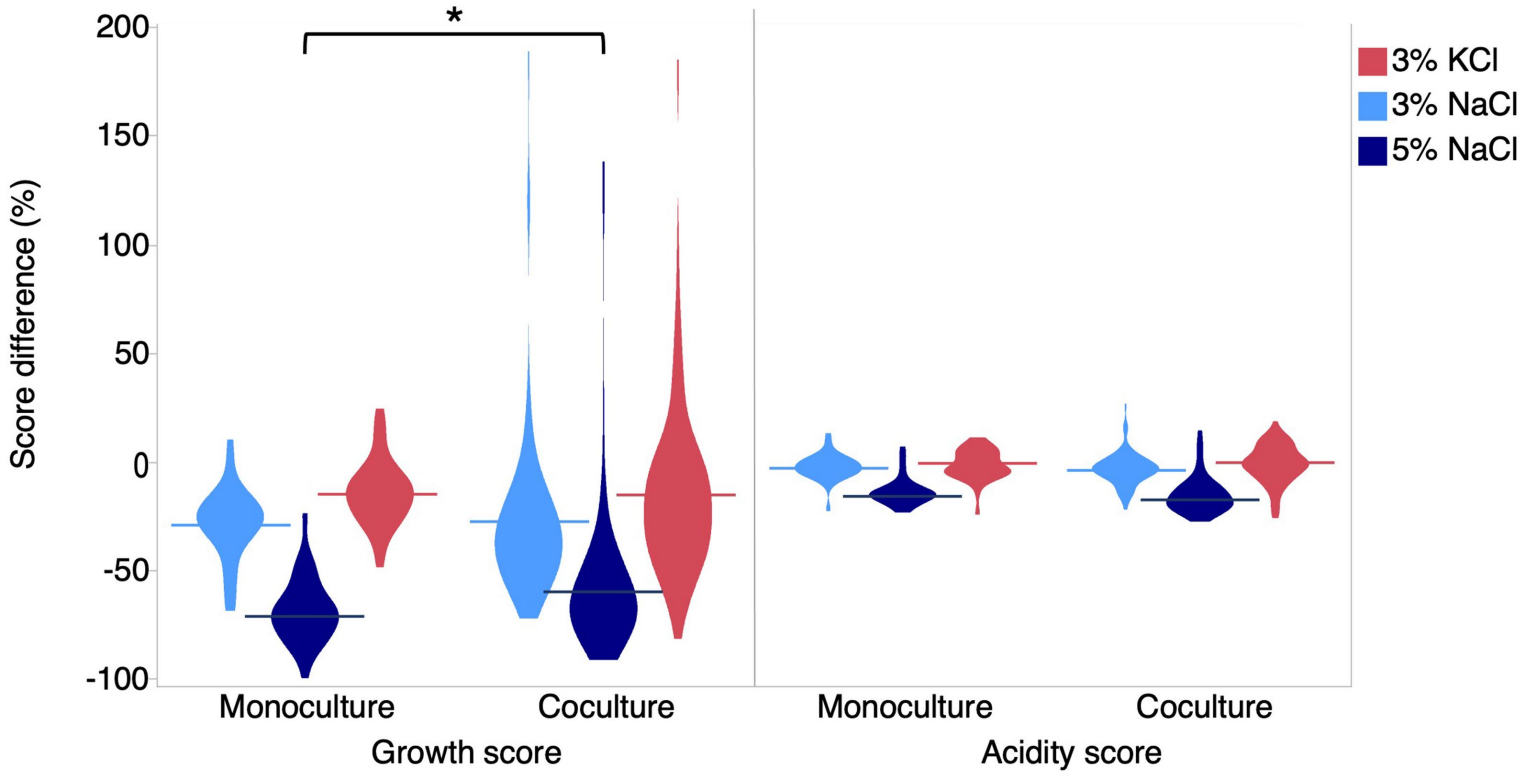
Overall effects of salts on growth and acidification in monoculture and co-culture. Difference of growth and acidification score for each salt condition. Light blue shapes represent the effect at 3% NaCl, dark blue shapes represent the effect at 5% NaCl, and red shapes represent the effect at 3% KCl. The asterisk symbolizes a significant difference between growth at 5% NaCl for co-cultures and growth for other conditions in monocultures.

To determine the effect of salt on LAB co-cultures, we randomly formed binary co-cultures between the 31 strains and measured their growth and acidification score in each condition. The co-culture growth scores were significantly lower than the control at 3% NaCl (*t*-test, 23.9%, *p* < 0.0001), 5% Nacl (*t*-test, 57.4%, *p* < 0.0001), and 3% KCl (*t*-test, 14.9%, *p* < 0.0001). However, for acidification, the reduction was significant only for 3% NaCl (*t*-test, 3.6%, *p* = 0.02) and 5% NaCl (*t*-test, 15.1%, *p* < 0.0001). Thus qualitatively, the salt effects on growth and acidification observed for co-cultures followed the same pattern as the results obtained for individual strains. Quantitatively, when comparing co-cultures with monocultures, we found that the variances for the growth score were unequal (Bartlett’s test <0.0001). Indeed, salt effects on co-culture growth scores were more variable than for monocultures, ranging from –89 to 186%. The average scores were not significantly different, except for 5% NaCl where the salt effect was weaker in co-cultures than in monocultures (Welch’s *t*-test, *p* < 0.05) (Figure 4, Supplementary Table 5). This result suggests that at higher concentrations, the growth of LAB co-cultures may be less impacted by salt effects compared to monocultures.

**Figure 5:**
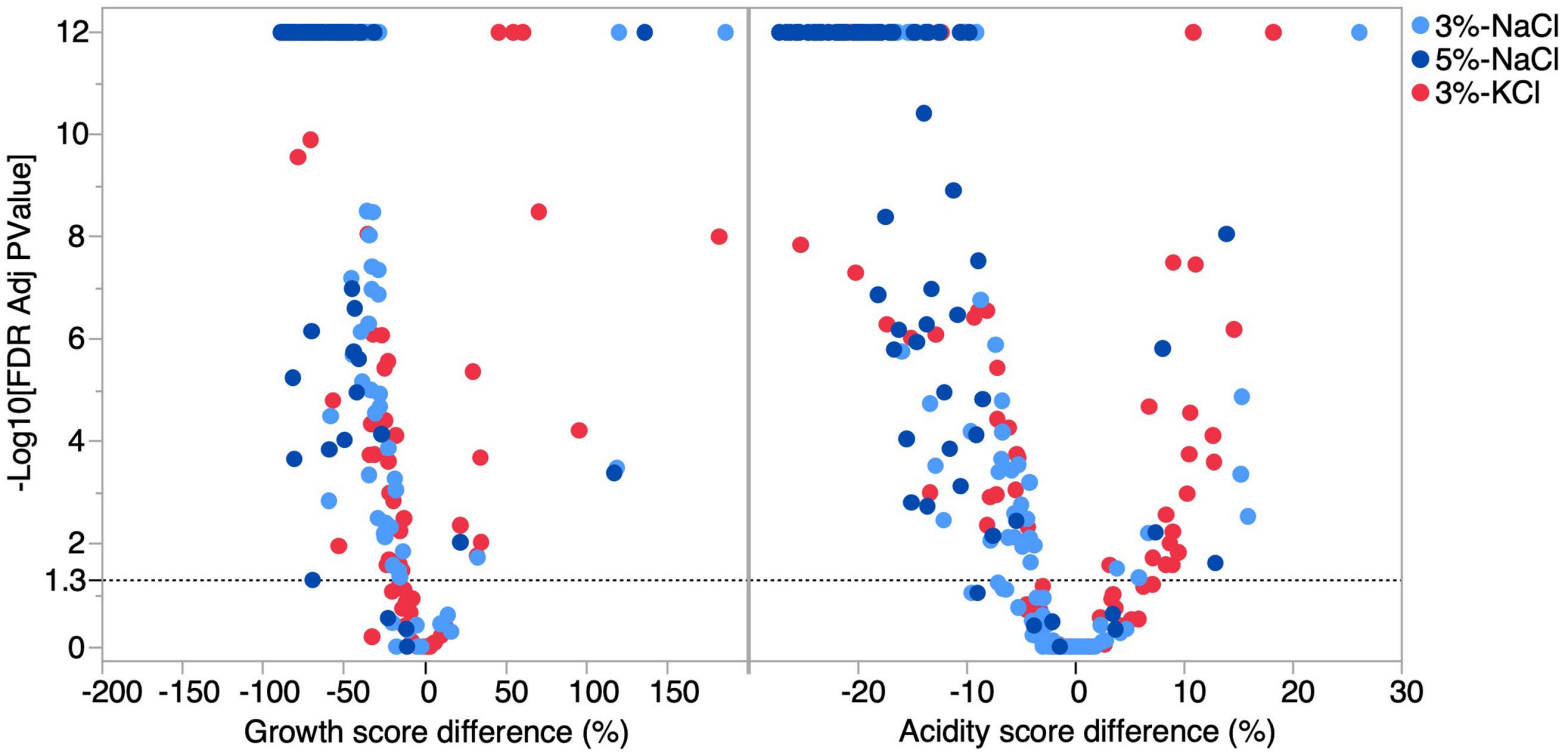
Volcano plot of the difference in growth and acidification score of co-cultures as a function of salts. The dashed line represents the significance threshold (FDR adjusted *p* = 0.05). Difference of growth and acidification score for each salt condition. Light blue circles represent the difference at 3% NaCl, the dark blue circles represent the difference at 5% NaCl, while the red circles represent the difference at 3% KCl.

Considering individual co-cultures, significant differences from the control condition were detected for growth and acidification scores in at least one salt condition for almost all the co-cultures (Dunnett’s test, FDR *p* < 0.05) (Figure 5, Supplementary Table 6). Although the general effects of salts were negative, cases of positive effects were detected for both the growth and acidification score. Positive effect cases were more abundant for 3% KCl but were also detected for 3 and 5% NaCl. For example, the co-culture formed by *Lactococcus raffinolactis* ATCC 43920 and *Lactococcus cremoris* 7311 showed an increase in growth of 117% at 5% NaCl (FDR *p* = 0.0004), 119% at 3% NaCl (FDR *p* = 0.0003), and 182% at 3% KCl (FDR *p* < 0.0001) compared to the control. Interestingly, the highest growth observed (186%) in co-cultures involved the same strain of *Lactococcus cremoris* 7311, but this time paired with *Lactococcus lactis* 74310.

Since salts can impact the growth and acidification of co-cultures, we asked whether the qualitative effect of salts (significant effects or not) on two monocultures could predict a significant effect on the growth and acidification outcome of their co-culture. In most cases, the effect of salts on monocultures could explain the result of the co-culture, but not always. Indeed, monocultures that were not sensitive to salts (non-significant effect) became so when in co-cultures (significant effect, induction cases). Cases where a significant effect was detected on both monocultures, but not on their co-culture (suppression cases) were also detected. In fact, for the growth score, suppression was more prevalent at 5% NaCl with 58.8% of the co-cultures, the co-cultures at 3% NaCl, 41.7% of co-cultures were affected, while only 5% of the co-cultures had lost their significant growth effect at 3% KCl. For acidification, 47.6% of co-cultures showed suppression at 5% NaCl followed by 3% NaCl by 38.1% of co-cultures and 25% of co-cultures at 3% KCl. Induction was significantly rarer than suppression, both for growth and acidification, although at 3% KCl, there were 13.9% of co-cultures with significant growth induction (Table 2). Altogether, these results suggest that co-cultures can buffer the effect of salts on LAB.

**Table 2:** Relative cases (%) of induction, suppression, and predictable cases in co-cultures for growth and acidification at different salt conditions.

	Acidity score			Growth score			
	3% KCl	3% NaCl	5% NaCl	3% KCl	3% NaCl	5% NaCl	*N*
Induction	3.6%	1.2%	2.4%	13.9%	0.0%	2.4%	19
Suppression	25.0%	38.1%	47.6%	6.3%	41.8%	58.5%	178
Predictable	71.4%	60.7%	50.0%	79.8%	58.2%	39.0%	293
Total							490

**Figure 6:**
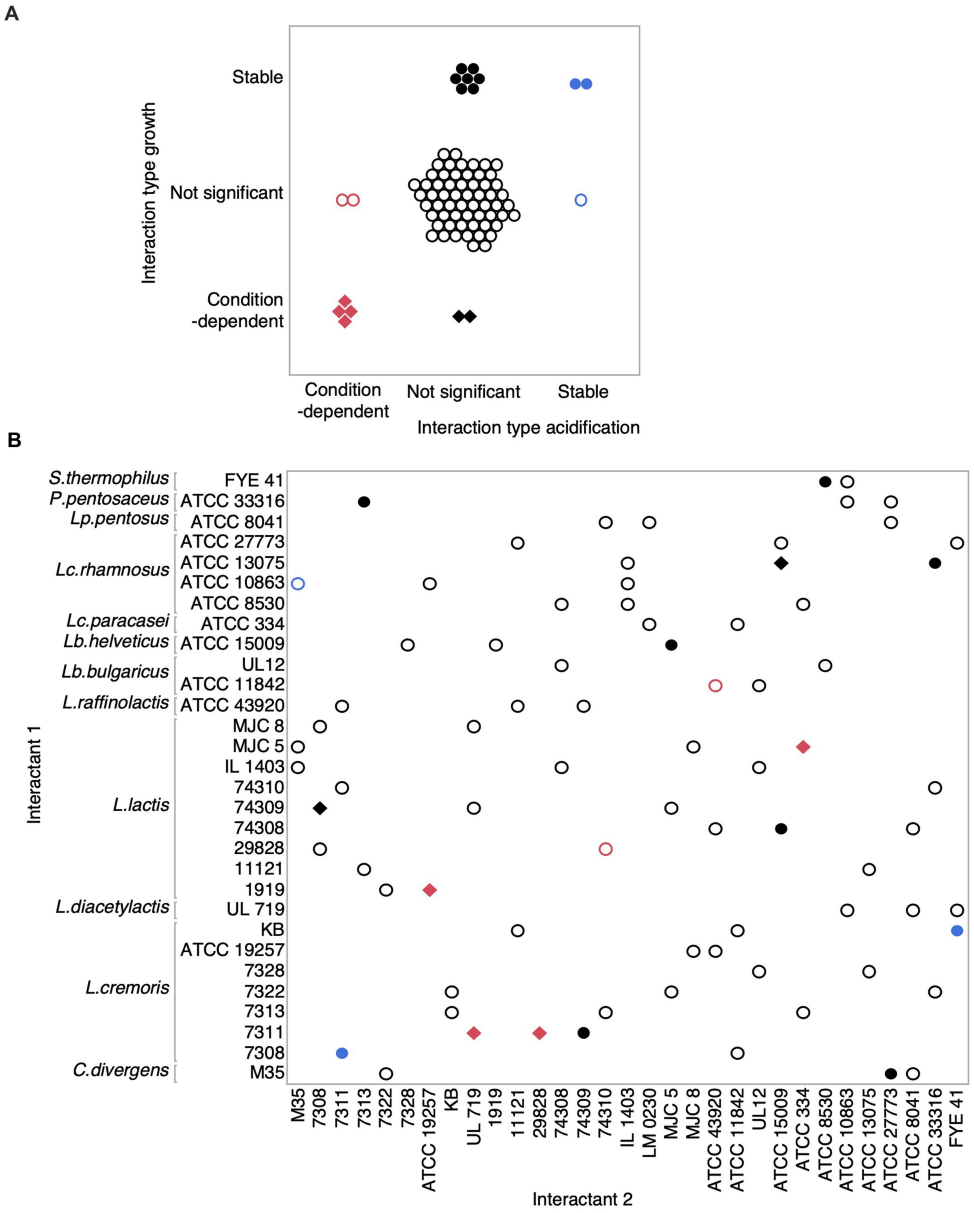
Microbial interactions measured on growth and acidification scores between lactic acid bacteria across salt conditions. **(A)** Overview of the intersection of detected effects on growth and acidification scores. Hollow circles represent either non-significant interactions for growth and acidification (black) or for one of these two measured parameters (blue and red). Solid circle points showed stable interactions for both growth and acidification (blue) or for one of these parameters (black). Diamonds represent condition-dependent interactions for growth and acidification (red) or for one of these parameters (black). **(B)** This graph shows the type of interactions between our LAB forming binary co-cultures. Colors and shapes match with those in **(A)**.

To assess how interactions between strains vary across salt conditions, we analyzed each microbial interaction based on the null hypothesis that co-culture average scores are not different from the overall mean of the individual strains. Microbial interactions were termed ‘condition dependent’ if the co-culture * salt interaction term was significant in the model and ‘stable’ if only the co-culture term was significant (Figure 6, Supplementary Table 7). Nine pairs (12%) for growth and two pairs (3%) for acidification showed stable microbial interactions. In fact, interactions stable for acidification were also stable for growth, i.e., the co-culture of *Lactococcus cremoris* KB and *Streptococcus thermophilus* FYE 41 and another co-culture formed by two *Lactococcus cremoris* (7308 and 7311). Condition-dependent microbial interactions were detected for six pairs (8%) and four pairs (5%) for growth and acidification scores, respectively. Thus, while co-cultures might buffer the effects of salts in some cases, salts can also influence some microbial interactions between LAB. Noticeably, the four condition-dependent microbial interactions detected for the acidification score were also significant for the growth score, indicating that some ecological interactions between LAB may be linked with metabolic strategies.

## Discussion

The effect of substituting NaCl with KCl for LAB in food products, although extensively studied (Hoffmann et al., 2019; Gan et al., 2021; Li et al., 2022) has mainly focused on a few species or only on total counts of LAB, thus overlooking the specific effects of salts on each strain. Similarly, the effect of salt stress on microbial interactions among LAB is not well understood in this context. In this study, we developed a high-throughput approach based on the use of a colony handling robot combined with image analysis to measure the growth and acidification parameters of individual strains. We showed that the growth and acidification capacity of LAB strains could be affected by the concentration and type of salt contained in the medium, namely NaCl and KCl. We tested two concentrations of NaCl, the most used salt, to illustrate that the concentration effect is measurable with our approach. We then tested KCl, which, according to the literature, is one of the most promising alternatives to NaCl. However, we limited our study to a low concentration of KCl because, at a certain concentration, KCl could impart a metallic taste to a food product, leading to rejection by consumers (Khetra et al., 2016). Moreover, we established that salts could modulate microbial interactions among LAB. In addition, we highlighted that co-cultures could attenuate the effect of salts on LAB. Furthermore, this study provides a novel dataset on acidification, growth, and interactions of several reference strains of LAB under salt stress.

Bacterial cell growth is not necessarily proportional to its acidification capacity, especially under stress conditions. The change in correlation between acidification and growth under salt conditions suggests that these parameters may be independently affected in some cases. Our findings are consistent with previous studies that have shown that the production of lactic acid is only partially associated with growth (Alvarez et al., 2010; Ghimire et al., 2020). For strains with low growth but relatively high acidification, as is the case for *Lactococcus lactis* subsp. *lactis strains* LM 0230 at 0% salt and *Lactococcus cremoris* ATCC 19257 at 3 and 5% NaCl, this phenomenon appears to be a physiological trade-off because the production of lactic acid eventually inhibits growth. This is highly relevant because the production of organic acids during fermentation in dairy products not only inhibits pathogenic and spoilage bacteria, but also controls the growth of LAB that are the producers (Punia Bangar et al., 2022). Additionally, certain proteins involved in the carbon metabolic pathways of *Lactobacillus sakei* were shown to be affected in the presence of NaCl during growth (Marceau et al., 2004; Chaillou et al., 2005). In our case, it is possible that a similar change in metabolism in the presence of NaCl (3 and 5%) has altered the correlation between growth and metabolism-related acid production.

Microorganisms are sensitive to changes in their environment. We have demonstrated that the concentration and type of salt affect the growth and acidification of LAB. While in several studies only total plate counts of LAB are considered (Laranjo et al., 2017; Zinno et al., 2017; Tidona et al., 2019; Juan et al., 2022), here we have evaluated the effect of salts on the individual growth and acidification of 31 LAB strains. Investigating the impact on each strain is relevant when selecting lactic ferments and probiotics because total counts can obscure distinctive patterns. This is even more true regarding food spoilage, which could be specific to a strain and cannot be collectively attributed to the species (Laursen et al., 2005; Leisner et al., 2007; Pothakos et al., 2015) Furthermore, genetic heterogeneity is higher at the strain level than at the species level, leading to variations in the response to environmental stresses (Erkus et al., 2013). Our results, in agreement with previous studies, suggest that KCl reduces the growth of the majority of our LAB strains, but not as much as NaCl (Karimi et al., 2012) and may not drastically alter their acidification capacity (Hystead et al., 2013). This could partly be explained by the fact that NaCl has a more significant impact on water activity than KCl (Arboatti et al., 2014; Zhang et al., 2020). In contrast, other studies have found that substituting NaCl with KCl did not affect the total growth of LAB but increased pH in Halloumi cheese (Kamleh et al., 2012) and in semi-hard cheese (Juan et al., 2022). However, our results clearly show that the effects of salts are strain dependent. Although we used a modified generic medium, it is important to emphasize that the results obtained could vary depending on the food matrices. In this regard, substituting NaCl with KCl should not be a generalized rule, because each food product has its own compositional and microbiological specificity that must be taken into account (Gagnaire et al., 2022; Tidona et al., 2022).

Using an automated robotic method and image analysis, we were able to capture and analyze both ecological (growth) and functional (acidification) interactions of binary combinations of strains under different salt conditions. Some microbial interactions differed between salt conditions. These results reveal that salts can influence microbial interactions among LAB at the ecological and functional levels, i.e., their growth and acidification. In fact, we demonstrated that significant growth or acidification effects were induced and suppressed in certain co-cultures, contrary to what was expected on the basis of the results from pure cultures. Suppression was highly recurrent in the presence of NaCl for both growth and acidification, and to a lesser extent for KCl. This result seems to indicate that cohabitation could allow LAB to cope better with stressful environments, such as the presence of salt. For instance, under monoculture conditions, we noticed that *Lactococcus cremoris* 7311 did not seem to be influenced by salt, which could explain the resilience among some of the co-cultures in which it is involved. The high variability in the response to salt stress in co-culture compared to that in monoculture may be explained by the interactions established between LAB in co-culture. This assertion is consistent with previous studies that have shown that interactions between bacteria can modulate their response, making them more resilient to salt stress (Bharti et al., 2015) or developing tolerance to an antibiotic (Pearl Mizrahi et al., 2023). It should be noted that our growth score represents the total biomass of a defined culture position on the plate that may contain exopolysaccharides known to protect against environmental stresses, including salt stress. Also, bacteria can establish microbial cell-to-cell interactions through exopolysaccharides to form stress-resistant biofilms (Dertli et al., 2015).

Among the co-cultures tested, relatively few microbial interactions were detected. However, our approach has demonstrated its effectiveness in identifying microbial interactions that involve *Lactococcus cremoris* and/or *Lactococcus lactis subsp. lactis*, two species that are traditionally used in combination as starter cultures during the manufacture of cheddar cheese (Poudel et al., 2022). Also, we detected context-dependent interactions (15% for growth and 8% for acidification). Microbial interactions are known to be context-dependent (Bittleston et al., 2020), but to which extent is often unknown. Here, we have demonstrated that with an abiotic gradient, namely salt, nearly half of the measured interactions changed depending on the context. It is also possible that other interactions existed, but our method may not be as sensitive to detect them. Furthermore, microbial interactions can occur at other levels, such as proteolytic activity (Chaves-Lopez et al., 2014) or at the molecular level with a modification of the gene expression profile (Ikeyama et al., 2020).

Our study was a proof of concept that we could monitor the growth and acidification of lactic bacteria and their co-cultures using an automated colony handling platform. So far, very few studies have employed this technology in the field of food microbial ecology (Blasche et al., 2021) despite its major contribution to study model organisms (Nichols et al., 2011; Costanzo et al., 2016; Koo et al., 2017) One limitation of our approach is that it does not allow us to capture the individual impact of the interaction on each of the strains involved, since only the overall net effect is measured. In the present context, acidification and growth of the co-culture are the parameters of interest, which do not require investigating the individual effects of the strains. However, in the context of studying interactions between LAB and undesirable microorganisms, it would be relevant to measure the effects on each strain of the co-culture. Further studies should address this challenge, as well as improving the automation of image analysis to minimize the requirements of manual input.

## Conclusion

The automated approach implemented allowed us to establish on a large scale that salt can modulate LAB growth and metabolism. Moreover, understanding LAB at the strain level, as well as the resulting microbial interactions, is essential for the successful use and control of LAB in food products. With the ability of co-cultures to buffer the effect of salts on LAB, our results reinforce the idea that co-cultures contribute to LAB resilience in stressful environments. However, further development of high-throughput methods that consider the net and individual effect on each microorganism involved in microbial interaction, especially when it comes to contaminants, is needed to better leverage their outcomes.

## Data availability statement

The data presented in the study are deposited in the Zenodo repository, accession number 10020400 (https://doi.org/10.5281/zenodo.10020400).

## Author contributions

AN: Conceptualization, Data curation, Formal analysis, Investigation, Methodology, Validation, Visualization, Writing – original draft, Writing – review & editing. IF: Conceptualization, Funding acquisition, Resources, Supervision, Writing – review & editing. MF: Conceptualization, Formal analysis, Funding acquisition, Methodology, Project administration, Resources, Supervision, Writing – review & editing, Validation.
